# Kinematic and Kinetic Comparison of Sprint-Specific Exercises: Impact on Maximal Sprint Acceleration Training

**DOI:** 10.5114/jhk/211899

**Published:** 2025-10-24

**Authors:** Jérémy Jusseaume, Charly Fornasier-Santos, Jean-Benoit Morin, Benjamin Millot, Gaël Guilhem, Jean Slawinski

**Affiliations:** 1 French National Institute of Sport, Expertise and Performance; Sport, Expertise and Performance Lab, Paris, France.; 2 Laboratoire Interuniversitaire de Biologie de la Motricité, Univ Lyon, UJM-Saint-Etienne, Saint Etienne, France.; 3 Performance Optimization Team, French Athletics Federation (FFA), Paris, France.

**Keywords:** mechanics, training, running, categorisation

## Abstract

Sprint-specific exercises (SSEs) are believed to train force, power and/or velocity qualities involved in the acceleration phase of sprinting. However, the kinetics and the kinematics of such exercises have never been explored. The aim of this study was to compare mechanical variables (horizontal and vertical forces, horizontal velocity of the centre of mass and the ratio of force) between SSEs and an all-out 40-m sprint acceleration (Sref). These variables were measured over each situation (Sref and 14 SSE) using six track-embedded force plates. The horizontal forces and velocities were either lower or equal to those of the Sref (SSE grand average deviation from FVP ~ −0.29 N∙kg^−1^ for force; SSE grand average from Sref ~ −0.14 m∙s^−1^ for velocity), while vertical force output was mostly greater in the SSE than the Sref (SSE mean deviation from Sref ~0.49 N∙kg^−1^). The ratio of force was lower or equal for the SSE compared to Sref. Despite large inter-individual variability, these SSEs seem useful to stimulate vertical force production, and not horizontal as hypothesised by coaches. These results suggest the importance of analysing the SSE used during training, from a force-velocity point of view, to better characterize their effectiveness.

## Introduction

High-velocity running is a fundamental component of many team and individual sports. The achievement of high velocity running mainly depends on the acceleration quality of the athlete (Rabita et al., 2015a). The acceleration phase can be divided into different successive phases: a start and initial acceleration followed by transitional acceleration followed by maximum velocity. Using the power velocity relationship, this acceleration phase can also be divided in two phases: a force-power phase and a power-velocity phase (Slawinski et al., 2022). The force-velocity and power-velocity profiles (F-v and P-v) of the acceleration phase determine the three main components of performance during the sprint: force, power, and velocity (Rabita et al., 2015b; Samozino et al., 2022; Slawinski et al., 2017). Sprint training programs aim to optimise these three components.

To prepare athletes to sprint, training programs usually use resistance training and set distinct objectives to develop force, power, and velocity, based on the findings of specific tests. For example, they may determine the athlete’s one-repetition maximum (1RM) and train them at a specific percentage of this value (Kraemer et al., 2017). Analysis of the lower limb force-velocity profile during movements such as the squat jump can be used to identify aspects which require training (Rahmani et al., 2001; Samozino et al., 2008). These evaluations can help determine the training contents, and exercises are chosen depending on their expected impact on the athlete’s strength development.

In a recent series of papers, Loturco and his colleagues (Loturco et al., 2023a, 2023b, 2023c, 2024) studied practices of Brazilian Olympic sprint and jump coaches. They demonstrated that training of Olympic athletes was a sophisticated blend of plyometric, speed, and resistance training. To develop sprint velocity, they used a wide range of speed training and methods. Another component of sprint training is the use of resistance. Using a sled-resistance, a slope, a parachute or equivalent electronic resistance systems, it is possible to modify the load that reduces (or increases) the maximal velocity of sprinting. These methods can be applied to train force, power, and velocity (Cross et al., 2017, 2018). Heavy loads (i.e., high resistance) are classically recommended to develop the force end of the spectrum, while intermediate loads are aimed at power enhancement and lighter loads are recommended for velocity (Cahill et al., 2019; Cross et al., 2017; Hicks et al., 2019). Resisted sprint work increases muscle activation, stride length and the production of horizontal forces (Cahill et al., 2020; Petrakos et al., 2016). In addition, overspeed training can also be used to develop maximal velocity (Lahti et al., 2020).

A well-designed, comprehensive sprint training program must also include sprint-specific exercises (SSEs: performed on a track with no additional load) that develop sprinting skills and technique (Hicks et al., 2022). A thorough understanding of the effects of these SSEs would enable them to be categorised and positioned according to the specificity principle of sprinting to ensure their effective use. In particular, SSEs have been little studied and no studies have yet determined which SSEs most effectively train and develop force, velocity and power. Measurement of the development of force and velocity during these exercises compared to an all-out 40-m sprint acceleration (Sref) would provide important knowledge of the mechanics of SSEs.

Therefore, the aim of this pilot study was to compare ground reaction force (production and orientation) and velocity output during 14 specific SSEs with an all-out 40-m sprint acceleration. Our overall aim was to increase understanding of training content to optimise training programs. As suggested by coaches, we hypothesized that these 14 specific SSEs would be more effective to improve the forward displacement of the athlete. Thus, greater horizontal force and a lower ratio of force should be observed for the SSEs.

## Methods

### Participants

Athletes with at least three years of track and field experience who competed at regional and national levels participated in the present study (n = 6; 3 males and 3 females). They were specialised in sprinting and/or hurdling and were well-accustomed to the SSEs studied (Table 1). Participants were instructed not to perform any strenuous exercise in the 24 h prior to the tests. None had experienced a lower limb injury in the three months preceding the tests. Participants were informed about the nature, aims and risks associated with the experimental procedures before providing written consent. This study was conducted following the principles of the Declaration of Helsinki, and approved by the Institutional Review Board of the CPP Ouest, Tours, France (approval code: ANR-19-STPH-0003; approval date: 11 February 2022).

**Table 1:** Participants’ characteristics. Values are means of all participants ± standard deviations.

	Track and field training experience (in years)		Age (in years)	Body mass (in kg)	Body height (in cm)	Best performance over the 40-m dash (in s)
Men (n = 3)		10 ± 4.9	23.4 ± 3.4	72.7 ± 5.0	178.3 ± 1.2	5.56 ± 0.09
Women (n = 3)		5.3 ± 2.6	19.9 ± 3.2	60.3 ± 3.3	169.0 ± 4.9	5.97 ± 0.16
All participants (n = 6)		7.7 ± 4.6	21.6 ± 3.8	66.5 ± 7.5	173.7 ± 5.9	5.76 ± 0.24

### Design and Procedures

This study was carried out in three steps. The first step involved identifying SSEs frequently used in sprint training. We interviewed four international and national level coaches about the exercises they used with their athletes. The selected exercises were then described according to the type of the start (i.e., initiated from a static position or with an initial velocity different from 0), the starting position (i.e., standing, tripod, quadrupedal), and the nature of the exercise (i.e., initial acceleration phase exercises, plyometric drills, top speed exercises etc.). From this first step, coaches selected 14 specific exercises (SSEs) that were representative of those typically used in training (Table 2).

**Table 2:**
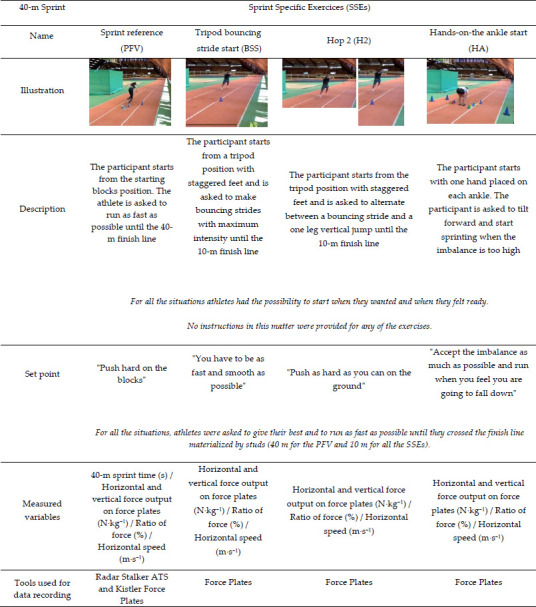
(Part 1). Summary table of the exercises carried out during the experiment (description and specific instruction).

**Table 2:**
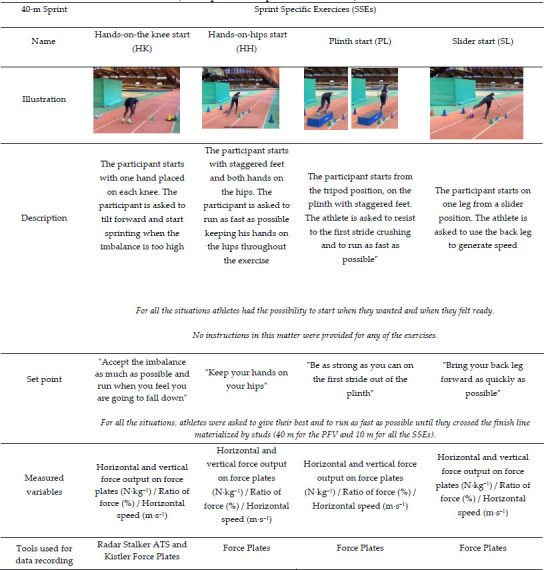
(Part 2). Summary table of the exercises carried out during the experiment (description and specific instruction).

**Table 2:**
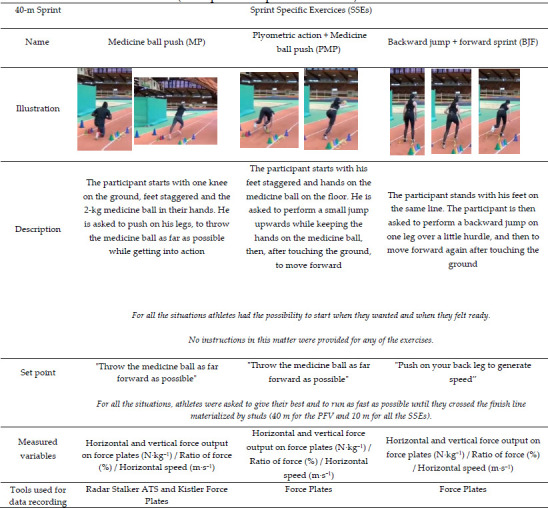
(Part 3). Summary table of the exercises carried out during the experiment (description and specific instruction).

**Table 2:**
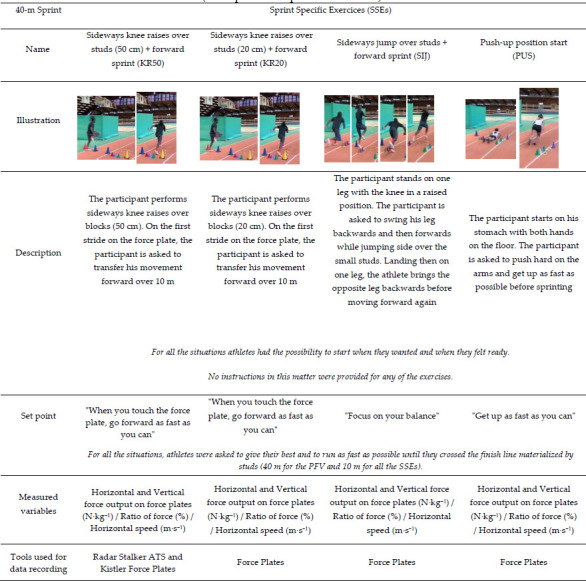
(Part 4). Summary table of the exercises carried out during the experiment (description and specific instruction).

The second step consisted of performing an all-out 40-m sprint acceleration (Sref). Each participant completed this sprint on an indoor athletic track during a standardised sprint session. After a 30- to 45-min warm-up managed by their coach, athletes performed two Sref trials (block start to simulate competition conditions) with 4 min of recovery in between. Six force plates connected in series embedded in the track (6 × 1.20 × 0.6 m; KI 9067; Kistler, Winterthur, Switzerland) (Slawinski et al., 2017) allowed to record the ground reaction forces and to calculate velocity developed by athletes from the starting block to the 6.6^th^ m of the sprint.

The third step consisted of measuring the ground reaction forces and velocity developed by athletes during each SSE during the first 6.6 m of the exercise allowing to record the first four to five steps of each SSE. The mean values were calculated for each step to avoid the effect of differences in the step number for each participant and each exercise: these values were then averaged to obtain the mean of all step values for each variable during the SSE. Each athlete performed a set of 14 different SSEs in randomised order over 10 m. Eleven SSEs involved a start on the force plates and three off the force plates. Each athlete performed two trials of each exercise; an additional trial was performed if the coach determined that the technical performance of the first two trials was incorrect. Thus, we recorded more than 120 steps for each athlete. Athletes were asked to perform each SSE as fast as possible. To ensure maximal performance, a minimal 3-min recovery was imposed between each exercise. SSEs and the Sref were filmed using a high frequency camera (1080 p; 240 Hz; IPAD pro, Apple). The video recordings were used to identify and discard trials during which a foot did not contact the force plates.

### Data Processing and Analysis

We considered two types of exercises: the exercises initiated from a static position with an initial velocity equal to 0 (Sref; BSS; H2; HA; HK; HH; PL; SL and MP; Table 2) and exercises with a flying start (initial velocity different from 0). For these latter exercises, the start was either off the force plates (KR50; KR20; SIJ; Table 2) or on the force plates but with an initial run-up movement prior to the actual performance of the SSE (PMP; BJF; PUS; Table 2).

Force plates data were processed using Origin software (Origin 2021). The experimenter identified touchdown and toe-off moments (using vertical axis force data: threshold set at 10 N) for every step recorded for each exercise. For each contact phase detected, the average horizontal and vertical components of the ground reaction force were computed as previously described (Slawinski et al., 2017). We then calculated the average ratio of force (RF, expressed in %) at each step according to the following equation:


$$RF_{\text{average}}\%=\frac{F_{y}}{\sqrt{F_{y}^{2}+F_{z}^{2}}}\times 100$$

For the exercises initiated from the static position, we calculated the average horizontal velocity at each contact phase in m∙s^−1^ as follows:


$${\text{V}}_{\text{yaverage}}=v_{0y}+\int_{t}A_{y}dt$$

where A_y_ corresponded to the horizontal acceleration of the centre of mass (COM) and V_0y_ corresponded to initial velocity (V_0y_ = 0). This expression was integrated once over time to determine the instantaneous horizontal velocity of the COM (V_y_) at time t.

For each exercise and every participant, the data from the four variables evaluated for each step over the 6.60-m force plates were then averaged (mean values of all steps computed for each variable during the SSE). We calculated the mean horizontal and mean vertical components of the ground reaction forces (F_ymean_ and F_zmean_ in N∙kg^−1^, respectively,) and the mean ratio of force (RF_mean_%) during the contact phases for each SSE and the Sref. For the SSE initiated from a static position, we also calculated the mean horizontal velocity of the centre of mass (V_ymean_ in m∙s^−1^).

### Statistical Analysis

We analysed F_ymean_, F_zmean_, RF_mean_% and V_ymean_ for all the SSEs and the Sref. The values obtained during the Sref over the 6.60-m force plates were considered reference values. We applied non-parametric tests for variables which did not follow a normal distribution for at least one exercise: we used a Friedman non-parametric test for F_ymean_, F_zmean_ and RF_mean_% to compare each SSE with the Sref, and a one-way ANOVA with repeated measures (exercise effect) for V_ymean_ to compare each SSE with the Sref.

## Results

### Sprint-Specific Exercises Selected for the Study

Out of the 175 exercises initially identified by coaches, we selected 14 SSEs (Table 2). The choice was made according to the exercises that were deemed most important by the coaches involved in the study and also if the exercises could be performed on force plates. All exercises were either initial acceleration phase exercises or initial acceleration phase exercises coupled with plyometric drills.

### Mean Horizontal Component of the Ground Reaction Force (Fymean N∙kg^−1^)

**Figure 1:**
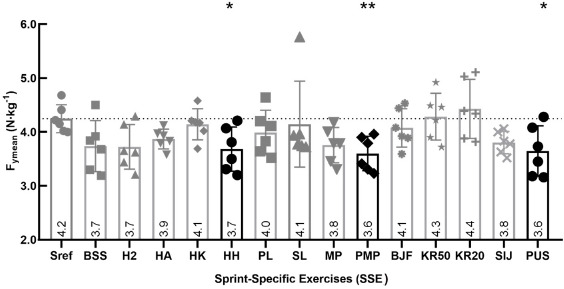
Comparison of mean horizontal force production (F_ymean_ N∙kg^−1^) between the SSEs and the Sref. Graph represents the mean horizontal force production over the 6.60 m of force plates (F_ymean_ N∙kg^−1^); values are means of all participants. In black, there are values that differed significantly between the SSE and the 40-m sprint reference (Sref); * significant difference at p < 0.05; ** significant difference at p < 0.01; the dotted line shows the number of participants for whom values during the SSE were above the Sref value; tripod bouncing stride start (BSS), hop 2 (H2), hands-on-the ankle start (HA), hands-on-the knee start (HK), hands-on-hips start (HH), plinth start (PL), slider start (SL), medicine ball push (MP), plyometric action + medicine ball push (PMP), backward jump + forward sprint (BJF), sideways knee raises over studs (50 cm) + forward sprint (KR50), sideways knee raises over studs (20 cm) + forward sprint (KR20), sideways jump over studs + forward sprint (SIJ) and push-up start (PUS)

F_ymean_ developed during HH, PMP and PUS exercises was significantly lower than F_ymean_ developed during the Sref (*p* < 0.05). These differences were respectively −11.9% for the HH, −14.3% for the PMP and 14.3% for the PUS exercise. F_ymean_ did not differ between the Sref and the other SSEs (*p* > 0.05) (Figure 1).

### Mean Vertical Component of the Ground Reaction Force (Fzmean N∙kg^−1^)

**Figure 2:**
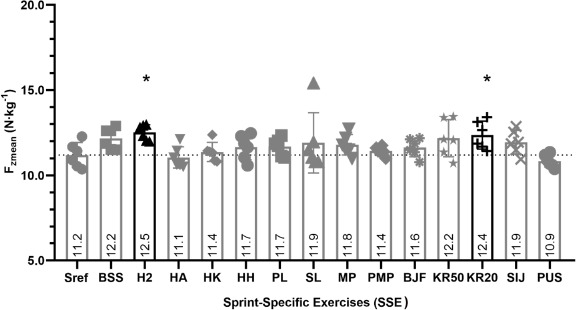
Comparison of mean vertical force production (F_zmean_ N∙kg^−1^) between the SSE and the Sref. Graph represents the mean vertical force production over the 6.60 m of force plates (F_zmean_ N∙kg^−1^); values are the means of all participants. In black, there are values that differed significantly between the SSE and the 40-m sprint reference (Sref); * significant difference at p < 0.05 with the Sref; the dotted line shows the number of participants for whom values during the SSE were above the Sref value; tripod bouncing stride start (BSS), hop 2 (H2), hands-on-the ankle start (HA), hands-on-the knee start (HK), hands-on-hips start (HH), plinth start (PL), slider start (SL), medicine ball push (MP), plyometric action + medicine ball push (PMP), backward jump + forward sprint (BJF), sideways knee raises over studs (50 cm) + forward sprint (KR50), sideways knee raises over studs (20 cm) + forward sprint (KR20), sideways jump over studs + forward sprint (SIJ) and push-up start (PUS)

F_zmean_ developed during H2 and KR20 exercises was significantly higher than F_zmean_ developed during the Sref (*p* < 0.05). These differences were respectively +11.6% for the H2 and +10.7% for the KR20 exercise. F_zmean_ did not differ between the Sref and the other SSEs (*p* > 0.05) (Figure 2).

### Mean Horizontal Velocity of the Centre of Mass (Vymean m∙s^−1^)

**Figure 3:**
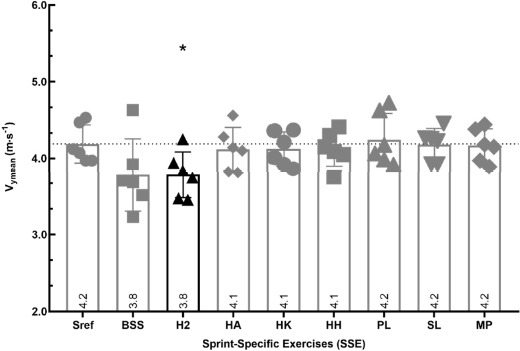
Comparison of mean horizontal velocity (V_ymean_ m∙s^−1^) between the SSE and the Sref. Graph represents the mean horizontal velocity over the 6.60 m of force plates (V_ymean_ m∙s^−1^); values are means of all participants. In black, there are values that differed significantly between the SSE and the 40-m sprint reference (Sref); *significant difference at p < 0.05; ** significant difference at p < 0.01 with the Sref; the dotted line shows the number of participants for whom values during the SSE were above the Sref value; tripod bouncing stride start (BSS), hop 2 (H2), hands-on-the ankle start (HA), hands-on-the knee start (HK), hands-on-hips start (HH), plinth start (PL), slider start (SL) and medicine ball push (MP)

V_ymean_ developed during the H2 (−9.5%; *p* = 0.0262 < 0.05) was significantly lower than V_ymean_ developed during the Sref (4.2 m∙s^−1^). These variables did not differ between the Sref and the other SSEs (*p* > 0.05) (Figure 3).

### Mean Ratio of Force (RF_mean_%)

**Figure 4:**
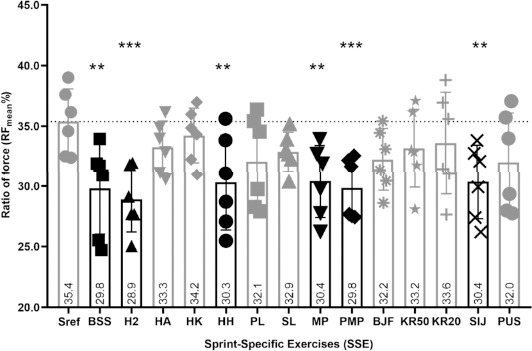
Comparison of the mean ratio of force (RF_mean_%) between the SSE and the Sref. Graph represents the mean ratio of force over the 6.60 m of force plates (RF_mean_%); values are means of all participants. In black, there are values that differed significantly between the SSE and the 40-m sprint reference- (Sref); ** significant difference at p < 0.01; *** significant difference at p < 0.0001 with the Sref; the dotted line shows the number of participants for whom values during the SSE were above the Sref value; tripod bouncing stride start (BSS), hop 2 (H2), hands-on-the ankle start (HA), hands-on-the knee start (HK), hands-on-hips start (HH), plinth start (PL), slider start (SL), medicine ball push (MP), plyometric action + medicine ball push (PMP), backward jump + forward sprint (BJF), sideways knee raises over studs (50 cm) + forward sprint (KR50), sideways knee raises over studs (20 cm) + forward sprint (KR20), sideways jump over studs + forward sprint (SIJ) and push-up start (PUS)

RF_mean_% developed during BSS, H2, HH, MP, PMP and SIJ exercises was significantly lower than RF_mean_% developed during the Sref (*p* < 0.01). These differences were −15.8% for the BSS, −18.4% for the H2, −14.4% for the HH, −14.1% for the MP, −15.8% for the PMP and −14.1% for the SIJ exercise. These variables did not differ between the Sref and the other SSEs (*p* > 0.05) (Figure 4).

## Discussion

The aim of this study was to compare force and velocity output during SSEs performed on a track with no additional load and an all-out 40-m sprint acceleration (Sref). Most of these exercises generated smaller or equal forces and velocities in the anteroposterior direction, and higher vertical forces than the Sref.

This study was inspired by the work of Hicks et al. (2019) who sought to provide guidance to coaches by discussing the potential “position” of such exercises on a force-velocity spectrum. They proposed that sprint-skill training should include strength exercises (e.g., back squats and hip thrusts [loads > 85 % 1 RM] to improve force), resistance track exercises (e.g., sled pulls, prowler sleds with different loads) and plyometric drills (e.g., countermovement jumps, box jumps, reactive jumps to improve maximal movement velocity).

The present study demonstrated that it was possible to compare sprint performance variables between different SSEs and a Sref using a track-embedded force plate system. More than half of the 14 SSEs evaluated (n = 8/14; 57%) differed significantly from the Sref for at least one of the variables analysed (F_ymean_, F_zmean_, RF_mean_% and V_ymean_). These data enhance understanding of horizontal and vertical forces and horizontal running velocities produced during SSEs and will be useful for coaches.

Horizontal force output (F_ymean_ N∙kg^−1^) was significantly lower than the Sref for three of the SSEs (HH, PMP, and PUS). This could be explained by a smaller amount of arm movement in these exercises in comparison with sprinting: the HH exercise was performed with the hands on the hips, the PMP exercise with the hands on a medicine ball (at least for part of the movement) and the PUS exercise with the hands on the floor (at least for part of the movement). This likely reduced the acceleration of the free segments in comparison with the other SSEs and the Sref. Acceleration of free segments directly impacts the overall acceleration and force generated at the centre of mass. The lack of use of the arms thus directly reduced F_ymean_ during the first steps of the sprint (Kugler and Janshen, 2010; Otsuka et al., 2016; Slawinski et al., 2010). In contrast, the SSE that involved arm movement did not differ from the Sref in terms of F_ymean_. All the SSEs were chosen by coaches because they were classically included in sprint training programs to develop horizontal force production and were believed to follow the training specificity principle. Compliance with this principle was confirmed by the force analysis that showed that these SSEs induced equal horizontal forces to those developed during the first steps of a maximal sprint acceleration. Thus, SSEs that involve arm movement stimulate the development of horizontal forces.

Vertical force production (F_zmean_ N∙kg^−1^) did not differ from the Sref for 12 SSEs and was significantly higher for two SSEs (H2 and KR20). This difference could be attributed to the rebound induced by the vertical one-leg jump in the H2 and the knee raise in the KR20 exercise. The SSE selected by coaches thus produced a F_zmean_ that was equal to or above that developed in a maximal sprint and therefore complied with the training specificity principle.

Six exercises were associated with a significantly lower mean ratio of force than the Sref (BSS, H2, HH, MP, PMP and SIJ exercises). One explanation for this is the overall increase in vertical force production and the slight decrease in horizontal force production in most of these SSEs compared with the Sref. The lower RF_mean_%, especially in the first few meters of the sprint, shows a decrease in the technical ability to orientate forces in the horizontal direction (Bezodis et al., 2021). This was expected for the BSS and the H2 because they are vertically oriented exercises. For the HH, the constrained arm movement may prevent appropriate force orientation. For MP, PMP and SIJ exercises, however, this result is surprising. These exercises involve throwing a medicine ball, which facilitates horizontal orientation of the body. Thus, it was expected that the RF would be greater than in the Sref. This demonstrates that the visual impression of horizontal orientation of the body does not actually indicate a higher RF. Consequently, practice of the BSS, H2, HH, MP, PMP and SIJ exercises may not improve the sprinter’s ability to direct forces in the horizontal direction. These exercises seem more appropriate to train the production of large vertical forces. They can, however, be used to develop overall bouncing and foot-ankle qualities by forcing athletes to resist intense impact and stance ground reaction forces, especially at high speed (Clark et al., 2017). We expected RF_mean_% to be greater in the eight other SSEs than in the Sref, however, there was no significant difference. Force orientation may, however, depend on the technical ability to perform these types of exercises and since the athletes who participated in the study were experienced in track and field training, the results of athletes with lower technical skills might have been different.

All the exercises were performed at the same mean horizontal velocity (V_ymean_) as the Sref, except for the H2. Mean horizontal velocity was lower for the H2 than the Sref. This could, at least partly, be explained by the vertical jump component of this exercise and the fact that athletes run effectively only with one leg. Together with the smaller horizontal forces produced during this exercise, this result suggests that the associated body displacement velocity of the H2 was sub-maximal compared to the Sref. We therefore suggest that this exercise should be used more for training technical skills rather than for the development of horizontal force and velocity qualities.

The 14 SSEs used in the present study were the most frequently applied by the track and field coaches interviewed, however, they may not be the most frequently used by all coaches. Future studies should complement the present results by evaluating other types of SSEs, for example, priming exercises performed between 2 and 48 hours before the competition phases (Pereira et al., 2025).

Some of the limitations of this study should be acknowledged. Substantial inter-individual differences were found in the responses to a given SSE (Table 3). This may be related to the small sample (N = 6), as well as the athletes’ maximal intention of the performance of each exercise, despite the instruction to perform all SSEs at maximal intensity. Moreover, although the technical performance of each exercise was verified by an expert coach, incorrect technique cannot be completely ruled out; this could lead to submaximal force and/or velocity output during the SSE compared to the Sref and constitute a confounding factor. Studies with larger samples are thus required to confirm the results.

**Table 3:** Inter-individual differences between all the participants each SSE. Values are means of all participants ± standard deviations.

Variables SSE	F_ymean_ (N∙kg^−1^) (N = 6)	F_zmean_ ( N∙kg^−1^) (N = 6)	V_ymean_ (m∙s^−1^) (N = 6)	RF_mean_ (%) (N = 6)
Sref *(40-m sprint)*	4.2 ± 0.3	11.2 ± 0.7	4.19 ± 0.25	35.4 ± 2.7
BSS *(Tripod bouncing stride start)*	3.7 ± 0.5	12.2 ± 0.6	3.78 ± 0.48	29.8 ± 3.8
H2 (Hop 2)	3.7 ± 0.4	12.5 ± 0.5	3.78 ± 0.30	28.9 ± 2.7
HA *(hands-on-ankle start)*	3.9 ± 0.2	11.1 ± 0.6	4.12 ± 0.29	33.3 ± 2.2
HK *(hands-on-knee start)*	4.1 ± 0.3	11.4 ± 0.6	4.12 ± 0.22	34.2 ± 2.3
HH *(hands-on-hips start)*	3.7 ± 0.4	11.7 ± 0.7	4.13 ± 0.23	30.3 ± 3.9
PL *(plinth start)*	4.0 ± 0.4	11.7 ± 0.5	4.24 ± 0.34	32.1 ± 3.8
SL *(slider start)*	4.1 ± 0.8	11.9 ± 1.8	4.18 ± 0.21	32.9 ± 1.6
MP *(medicine ball push)*	3.8 ± 0.3	11.8 ± 0.6	4.17 ± 0.22	30.4 ± 3.0
PMP *(plyometric + medicine ball push)*	3.6 ± 0.3	11.4 ± 0.3		29.8 ± 2.5
BJF *(backward jump)*	4.1 ± 0.4	11.6 ± 0.5		32.2 ± 2.6
KR50 *(sideways knee; 50 cm)*	4.3 ± 0.4	12.2 ± 1.1		33.2 ± 3.2
KR20 *(sideways knee; 20 cm)*	4.4 ± 0.5	12.4 ± 0.8		33.6 ± 4.2
SIJ *(sideways jump)*	3.8 ± 0.2	11.9 ± 0.7		30.4 ± 3.1
PUS *(push-up start)*	3.6 ± 0.5	10.9 ± 0.4		32.0 ± 4.1

40-m sprint reference (Sref), tripod bouncing stride start (BSS), hop 2 (H2), hands-on-the ankle start (HA), hands-on-the knee start (HK), hands-on-hips start (HH), plinth start (PL), slider start (SL), medicine ball push (MP), plyometric action + medicine ball push (PMP), backward jump + forward sprint (BJF), sideways knee raises over studs (50 cm) + forward sprint (KR50), sideways knee raises over studs (20 cm) + forward sprint (KR20), sideways jump over studs + forward sprint (SIJ) and push-up start (PUS)

## Conclusions

The analysis of the forces and velocities generated during 14 different sprint-specific exercises and comparison with a Sref generated useful data related to the mechanical properties of these exercises. Contrary to what the coaches thought, these selected SSE did not generate higher horizontal forces. Most of the SSEs evaluated in this study generated smaller or equal horizontal forces and velocities and higher or equal vertical forces than the Sref. These results can be used to guide coaches in the implementation of exercises within training programs and may be used by researchers to further increase knowledge of sprint training. Future studies should include surface EMG measurements of the sequence and amplitude of muscle activity to improve understanding of muscle coordination and neuromuscular adaptations during different SSEs: these which would be useful for both training and injury prevention (Vigotsky et al., 2018).
